# Obligated To Say “Yes”: The How and Why Behind Transfer Decisions in Moribund Patients

**DOI:** 10.5811/westjem.48985

**Published:** 2026-03-02

**Authors:** Alexa Stefanko, Nellie Trenga-Schein, Laura Chess, Mackenzie Cook

**Affiliations:** * Oregon Health and Sciences University, Division of Trauma, Critical Care and Acute Care Surgery, Portland, Oregon; † University of Vermont, Department of Emergency Medicine, Burlington, Vermont; ‡ Oregon Health and Sciences University, Department of Emergency Medicine, Portland, Oregon

## Abstract

**Introduction:**

A core principle of emergency care is the rapid transport of severely injured patients to hospitals capable of providing definitive care. Although the social, financial, and emotional factors associated with transfers, and their impact on hospital crowding, may necessitate a more nuanced approach, little has been published on how physicians actually make the decision to transfer a potentially moribund patient. We, therefore, sought to better understand these factors as the next step toward optimizing transfer flow and patient care.

**Methods:**

We conducted one-hour, semi-structured interviews with 16 emergency physicians at referring and referral centers, including eight accepting physicians at a quaternary-care center and eight transferring physicians at community hospitals. Interviews focused on decision-making regarding interhospital transfers for moribund patients, defined as those with injuries or disease processes judged likely to be non-survivable. Interviews were transcribed and analyzed using reflexive thematic analysis to identify common themes and decision-making factors.

**Results:**

We identified four emerging themes that underpinned a decision to transfer or accept a potentially moribund trauma patient: 1) the accepting physician’s perceived obligation to hospitals with fewer resources; 2) the difficulty of prognostication; 3) the imperfection and limitations of current advanced care planning documents; and 4) the impact of family and patient preferences.

**Conclusion:**

The rationale behind initiating and accepting transfers of moribund trauma patients is multifaceted. This study is the first to our knowledge that explores physician decision-making in this domain. Physicians feel an obligation to patients, families, and other hospitals, which leads to almost universally initiating or accepting transfers even in cases with limited hope of survival. These interviews offer insight into opportunities to improve statewide trauma operations and highlight avenues for promoting transfer-decision heuristics and pre-transfer goals-of-care conversations without compromising patient care.

## INTRODUCTION

A core principle of emergency care is the rapid transport of severely injured or ill patients to hospitals capable of providing definitive care. As the emergency department (ED) is often the first point of contact for critically ill and injured patients, emergency physicians must rapidly assess appropriate disposition within the constraints posed by time, incomplete clinical picture, and competing demand for hospital resources. Although transfer is often pursued with the goal of providing higher level care, regardless of available resources, the benefits of transfer become less clear in cases where survival is unlikely. Transfers can expose patients and families to substantial burdens, including emotional distress, social displacement, and financial strain, particularly on rural families.^1^–^3^

Transfers also rely on limited and valuable transportation resources such as fixed-wing aircraft and helicopters, which are unavailable for other rural emergency medical services while they are in use for a transfer. Prior research has found that 1.1–1.5% of interhospital transfers may be considered futile—defined as a hospital length of stay after transfer < 48 hours—and an interhospital transfer is estimated to cost approximately US $56,396.^4^,^5^ These challenges necessitate reexamining default transfer practices, particularly when aggressive intervention may not meaningfully alter outcomes. Despite the clinical, ethical, and logistical implications of transferring patients with poor prognoses, little is known about how emergency physicians navigate these decisions. Prior work has shown that explicit goals-of-care conversations occurred in only 10% of cases when trauma surgeons considered transferring moribund patients.^6^ However, limited information exists about the individual thought processes, concerns, and values that underlie decisions to initiate a potentially non-beneficial transfer.

With essentially no published literature on physician decision-making with regard to potentially non-beneficial transfers, we started with a qualitative, hypothesis-generating approach. We were specifically interested in the decision-making of emergency physicians when considering transfers for potentially moribund trauma patients. Our objective was to use semi-structured interviews to identify the common themes, challenges, and thought processes that influence emergency physicians’ decisions to transfer potentially moribund trauma patients.

## METHODS

### Study Design

Given the limited literature on this topic, we began with a qualitative approach that incorporated elements of phenomenology and the lived experiences of emergency physicians. The study underwent review by the institutional review board and was deemed to be exempt.

### Recruitment of Participants

A purposive sample of 87 potential participants—all of whom were emergency physicians practicing primarily in Oregon—were contacted via email; one-hour virtual interviews were arranged with those who consented to participate. We aimed to conduct a total of 16 interviews, split evenly between physicians at our quaternary-care center (accepting physicians), and physicians at community hospitals who transferred to our center (referring physicians). All but one of the community hospitals in our study operate independently and are not affiliated with the quaternary-care center. Our center prioritizes transfers from the hospital it owns. While six referring physicians also serve (or have served) as accepting physicians, we asked them to respond in their capacity as referring physicians only. We selected a target of eight participants per group based on prior research indicating that 6–12 interviews are typically sufficient to identify major themes in relatively homogenous populations.^7^ Once eight interviewees from each subgroup were enrolled, no further invitations were issued. Interviews took place between February–October 2023. Interviewees were not compensated for their participation.

**Population Health Research Capsule**

What do we already know about this issue?

*Futile transfers occur in 1.1–1.5% of cases and strain resources, yet little is known about how physicians decide on moribund transfers*.

What was the research question?

*What factors do emergency physicians consider when deciding to transfer moribund patients?*

What was the major finding of the study?

*Four themes guide transfer decisions: obligation to hospitals with fewer resources; difficulty of prognostication; limitations of advance care planning documents; and family preferences*.

How does this improve population health?

*Understanding these decision drivers can support goals-of-care discussions and align transfer decisions with patient values*.

### Data Collection

The semi-structured interview guides (appendices A and B) focused on the transfer decision-making process for moribund patients and perspectives on implementing statewide guidance for those making transfer decisions. Moribund was defined as having injuries or disease processes that were likely non-survivable, acknowledging inherent uncertainty in prognostication. After discussion with senior authors MC and LC, AS and NT drafted the interview guide based on clinical questions arising from their experience with interhospital transfers. After a final draft was prepared, a pilot interview was conducted, and minor adjustments were subsequently made to the draft for clarity.

Multiple authors (NT, AS, LC) initially participated in the interview process to calibrate the interviews. Neither author who participated in conducting interviews had a pre-existing relationship as colleagues or supervisors with any of the interviewees. Interviewers introduced themselves briefly at the beginning of each interview by stating their name and professional background. All interviewers were from the quaternary-care center. After completing three interviews as a team, AS transcribed and then coded the rest of the interviews.

### Analysis

Interviews were anonymized and uploaded to Dedoose analytic software (Sociocultural Research Consultants, LLC, Manhattan Beach, CA). Two researchers (AS, NT) independently conducted reflexive thematic analysis^8^ on two interviews, using an iterative approach to capture emergent themes. Subsequent interviews were conducted and coded by one researcher (AS). Coded interviews were discussed between multiple authors (AS, NT), discrepancies were addressed, and sub-codes were created. Areas of contradiction between interviewees were highlighted by notes and memos and then resolved during meetings. Thematic analysis proceeded according to Braun and Clarke’s six-phase framework: 1) familiarization with the data; 2) initial code generation; 3) theme generation; 4) theme review; 5) theme definition and naming; and 6) writing and interpretation.^8^

We considered thematic analysis to be complete when no new codes or concepts emerged from the interviews. Transcripts were not returned to the participants for comment. Researcher positionality was explicitly acknowledged: MC is a White, male allopathic physician and clerkship director; AS is a White, female medical student; NT is a White, female medical student, and LC is a White, female allopathic physician and an ED medical director. We used the Consolidated Criteria for Reporting Qualitative Research guidelines to ensure proper reporting of methods, results, and discussion.

## RESULTS

A total of 16 interviews were conducted: eight with emergency physicians at our quaternary-care center who receive transfers (accepting physicians) and eight with community emergency physicians who initiate transfers to higher levels of care (referring physicians). One accepting physician was primarily a pediatric emergency physician; the other seven accepting physicians saw primarily adults. All referring physicians saw both adults and children. Five interviewees were women, and six were men. Years of practice ranged from 2–36 years, with a mean of 16 years. Four of the community emergency physicians practiced outside the Portland metro area at the time of interview, and three worked at critical access hospitals. All practiced at trauma centers: three at Level II; three at Level III; and two at Level IV facilities.

All transfer calls were coordinated through a central transfer center. Trauma surgeons and neurosurgeons were rarely directly involved in transfer calls; instead, referring physicians usually communicated with accepting emergency physicians rather than subspecialists. Patients were almost always transferred to another ED for further workup, rather than the intensive care unit (ICU). The decision for a patient to be transferred to the ED vs directly to the ICU was driven by need for further evaluation, bed availability, and institutional capacity.

Key themes included the following: 1) “accepting physicians’ perceived obligation to hospitals with fewer resources”; 2) “difficulties with prognostication”; 3) “the limitations of current advance care planning documents”; and (4) “the impact of family and patient preferences” (Table). There was no disagreement regarding code application between the author who coded the interviews (AS) and the author who then reviewed those codes (NT). Each of these themes is described in the sections below with relevant quotes from interviewees.

### Accepting Physicians’ Perceived Obligation to Hospitals with Fewer Resources

Accepting physicians commonly reported feeling a sense of obligation toward referring hospitals within their region. They emphasized that larger centers were better equipped, both in staffing and technological resources, to handle complex cases. Accepting physicians saw their center as occupying a certain role in the state’s hospital ecosystem; quaternary-care centers are referral centers by nature and a resource for referring physicians at community hospitals who have reached the limits of their scope, resources, or expertise.

#### (Accepting physician 8)

For the most part, being where we are, we try to accept whatever comes, because we know what it is like to be the one that’s trying to do what’s right for the patient. And in the modern era, where capacity is truly an issue, that is challenging. But I still think it’s the right thing that if someone can’t handle something, to send it to us because we are supposed to be the hospital that can handle those things.

Accepting physicians also endorsed a fear of breaking trust between referring hospitals and accepting centers. They opted to accept transfers to maintain the trust of referring physicians and the flow of patients through escalating care centers.

#### (Accepting physician 5)

When you’ve got a physician at the other end and you say no... then, next time they have a transfer they want to send to you, they won’t send it to you. They’ll send it to somebody that didn’t say no. And that’s the reality. So, my philosophy is, I try to put myself in the person’s shoes... Blocking that transfer is not going to help them.

### Difficulties with Prognostication

Patient status and mortality risk are subjective and can fluctuate significantly in emergent situations, making prognostication challenging for participating clinicians. In the absence of a definitive outcome for a patient, accepting clinicians preferred to accept transfers to evaluate the patient for themselves, using the specialist and technological capabilities of their hospital. This was especially true when there was disagreement between referring and accepting physicians. Accepting physicians usually opted to err on the side of caution and accept such transfers, rather than risk a critically ill patient’s access to life-saving care.

#### (Accepting physician 5)

I think that the definition of non-survivable is difficult to determine because it’s a judgment call that’s made. You have to put yourself in the position of someone who is taken to a small hospital in the state, and then basically needs to go to a higher level of care. The issue of survivability is not easily determined initially.

#### (Accepting physician 6)

It’s hard to tell what is going to be non-survivable when you’re on the phone. We get the call when the patient is still alive. From a high-level view, I generally assume the person calling me recognizes that a) this person could be super sick or is very sick, b) regardless of severity they just might not be comfortable managing that patient, c) even if they’re not critically ill and that individual provider is comfortable, they just might not have the resources ... In the past, and still generally, I accept these calls with the default that I’ll accept the patient.

Referring physicians similarly endorsed the difficulty associated with prognostication, particularly in a resource-limited context that might have minimal support from other physicians or specialists.

#### (Referring physician 5)

There are a couple things that you have to think about, and one is the confidence that it is indeed non-survivable. And without any specialist, that can be difficult, because you don’t have anyone that can come down and give their thoughts. You’re usually the only person at bedside with that patient.

### The Limitations of Current Advance Care Planning Documents

Although increased use of advance care planning documents, such as physician orders for life-sustaining treatment (POLST) forms, is often discussed as a strategy to reduce futile interventions, both accepting and referring physicians described significant limitations. Participants noted that advance care documents frequently failed to anticipate unexpected clinical deterioration and were often not nuanced enough to guide complex transfer decisions.

#### (Accepting physician 5)

A POLST can be changed at any time ... You might have someone who has underlying dementia and has been in a care facility for the last four years. Today, they trip and fall and hit their head and now have a head bleed. Does that mean that nothing should be done? I don’t think so. I think that’s a misinterpretation of the POLST.

#### (Accepting physician 3)

[A patient] had underlying Parkinson’s disease. She had a POLST form, and then she choked. She suddenly couldn’t breathe, and then he tried to do the Heimlich, and he tried to do CPR, and then we arrived. And he said, “I want you to do everything to save her.” We basically threw out the POLST form and did what we could to try to resuscitate her.

Uncertainty also arose when healthcare proxies were unaware of the patient’s documented wishes and attempted to make decisions for an ill family member in unanticipated circumstances.

#### (Referring physician 4)

With POLSTS, a) you need to make sure it’s valid, b) we need to make sure the family knows it exists and where it came from and what’s going on with it. I frequently find that POLSTs get really mushy and flexible when people are under extreme decision-making capacity for an ill family member.

### The Impact of Patient and Family Preferences

Patient and family preferences played a major role in transfer decision-making. Both accepting and referring physicians described deferring to family wishes, even when the clinical team believed transfer might not substantially alter the patient’s outcome.

#### (Referring physician 2)

Ultimately, I’ll let the family make whatever decision is right for them and act on that. If a family demands transfer, if they demand the full court press, then I’ll try and get there.

Physicians, particularly those in more resource-limited areas, emphasized the importance of transparency around hospital capabilities and the likelihood of recovery. Occasionally, this led families to decline a transfer.

#### (Referring physician 4)

And the family said, “The most important thing for us is to keep him comfortable; can you keep him here?” .... We try to be really honest with what we can do and what’s available. I’m often surprised at how understanding and resourceful families are with the limitations that we’re stuck with.

The above theme regarding the limitations of advance care documents intersected with family preferences in meaningful ways. Physician motivations for considering patient preferences varied. Some emphasized the importance of creating a good death experience for the family, or expressed concern over litigation should the physician prioritize the planning document over the family’s preferences.

#### (Accepting physician 3)

That is an example of something where you could have said, “I’m not going to do anything.” Had you done that...it would have created a very negative death experience. And I think that—like the birth experience needs to be a valuable experience—I think the death experience needs to be one in which there is some closure.

#### (Referring physician 3)

You have to defer to the people that are alive and there and with it. You have to ignore that form. Because whether mom lives or dies, the person who’s gonna sue you is the one sitting next to you. And if they’re not happy and mom’s dead, and they blame you for not doing everything you could, even if you had the form, it would be a hard case to sell.

### Statewide Guidelines

All the participating physicians were asked for their perspectives on statewide guidelines to assist with transfer decision-making. While most (13/16) were in favor of such guidelines, many expressed caution in creating overly prescriptive tools designed to replace physician decision-making. One physician was explicitly opposed to such guidelines for this reason.

#### (Accepting physician 7)

I think having guidelines is great. However, the reality is that a lot of the patients you see don’t fit neatly into the guidelines that are created. So, it’s important that anything like that will always be a framework in which to work, allowing for clinician judgment for the patient in front of you.

#### (Referring physician 5)

I find that protocols in this type of thing are spectacular, because then the book doesn’t have to be re-written every time it happens … with the understanding that guidelines are guidelines, and it’s impossible to make a protocol that covers every single situation. So, there will at times be situations that diverge from protocols. But yes, it’s nice to have some standardization.

#### (Accepting physician 4)

This is like “we’re going to try to standardize care,” which I think is b******. I don’t think it’s meaningful. People want to try and introduce a level of predictability to medical care that is simply not going to happen. They want to try and introduce this idea that data will tell us how to manage individuals.

## DISCUSSION

This qualitative study provides insight into how emergency physicians navigate transfer decision-making for patients who may be moribund or have an exceedingly poor prognosis. A 2020 gap analysis conducted by the Critical Care Committee of the American Association for the Surgery of Trauma highlighted improving goals-of-care conversations within acute care settings as a top priority for future research.^9^ Notably, the analysis also found that end-of-life and goals-of-care discussions were among the least studied areas in critical care. Our findings contribute to this literature by indicating that, due to clinical uncertainty and the impact of factors such as family preferences, transfers are near-universally initiated and accepted, even when there is a mutual understanding that transfer is unlikely to significantly alter the patient’s medical trajectory. This extends prior work that demonstrates that even when the severity of the medical situation is known, physicians rarely discuss goals of care or code status.

While this decision appears to be driven by a desire to provide patients with access to potentially lifesaving care, it can expose patients and families to emotional, financial, and logistical burdens with the potential of not altering treatment outcomes. There is an opportunity to improve pre-transfer communication, particularly around goals of care, to ensure that transfer decisions are informed by likely clinical outcomes and patient and family values, considering those potential outcomes. However, we do not suggest that reducing transfers should be the sole aim; in fact, this study highlights the potential harm that a blanket restriction could cause to professional relationships, hospital referral networks, and clinicians’ moral compasses. Prior research has found that while inter-hospital transfer is associated with higher costs and longer length of stay, it also reduces the likelihood of 30-day mortality depending on the eventual diagnosis.^10^ Clinical uncertainty is often unavoidable, and transfer is often an appropriate course of action. When considering the decision to transfer, the goal is to better support clinicians in making these transfer determinations by offering frameworks and resources that augment clinical judgment.

### Accepting Physicians’ Perceived Obligation to Hospitals with Fewer Resources

Accepting physicians expressed that they felt an obligation to support clinicians and patients at referring hospitals, viewing transfer acceptance as central to their role as clinicians in a quaternary-care center. Some physicians were also concerned that declining a transfer could damage relationships between hospitals. This dynamic invokes prior findings that informal arrangements were more likely to dictate which hospital a patient was transferred to, rather than which hospital would necessarily fit the patient’s needs.^11^ It also reflects perceived and actual resource limitations in rural hospitals; for example, only 49% of critical access hospitals were found to have palliative care support, compared to 85% of non-critical access hospitals.^12^ Further investigation into these resource limitations, and whether transfer might be appropriate to secure a peaceful death, is warranted.

Additionally, what it means to be a good partner and quaternary-care center might benefit from reframing. Rather than defaulting to physical transfer, accepting hospitals could emphasize their role as clinical partners: supporting referring physicians through real-time consultations; providing guidance on prognostication; and, particularly, assisting with goals-of-care conversations.

### Difficulties with Prognostication

Both referring and accepting physicians described prognostication as a factor that contributed to the default to transfer. In the face of clinical uncertainty, physicians tended to favor accepting transfers to avoid denying potentially lifesaving care. Improving timely access to consultations may help clinicians navigate this uncertainty. Prior studies have emphasized the value of incorporating palliative care consults in the ED,^13^ and some centers reported positive results from including embedded palliative-care teams in the ED during the COVID-19 pandemic.^14^ Our findings suggest two complementary areas for improvement. First, improved access to telemedicine consultations and real-time data sharing could strengthen prognostic confidence by allowing emergency physicians to obtain timely second opinions or consults. This could be particularly useful for consulting palliative care remotely, which was employed to good effect in EDs during the COVID-19 pandemic.^15^ Second, clinicians can engage families in more structured discussions about goals of care and likely clinical trajectories while acknowledging uncertainty.

### Limitations of Current Advance Care Planning Documents

Physicians reported that existing advance care planning tools, such as POLST forms, were sometimes insufficient in the context of emergent transfer decisions, as they lacked nuance in situations that represented a significant change in a patient’s clinical state. This aligns with existing research that indicates POLST-discordant care is more common in the acute care setting^16^ and that fewer than half of POLST forms were concordant with current patient preferences.^17^ Addressing transfer preferences explicitly on these tools and ensuring that emergency physicians revisit goals-of-care conversations to account for a patient’s current condition may better inform transfer decisions in acute settings.

### Impact of Patient and Family Preferences

Physicians reported that the patient’s and family’s wishes significantly influenced transfer decisions, sometimes overriding concerns about the futility of a transfer. Physicians prioritized family autonomy, even when transfer was unlikely to change health outcomes. Clarifying family preferences is essential, as physicians otherwise may interpret a situation through their own biases. For example, a prior study of rural families during inter-hospital transfer found that physicians overestimated the importance of receiving care near a patient’s home and underestimated the patient’s desire to receive treatment in a comprehensive medical center.^18^

In our study, physicians appeared to recognize the patient’s and family’s desires to access definitive treatment above all else, facilitating transfers even when they questioned the likely clinical benefit, to honor those wishes. Early goals-of-care conversations can help ensure that treatment decisions are guided by patient- and family-centered values, rather than assumptions about proximity or intervention. These conversations must be approached carefully to avoid signaling that the care team is overly focused on end-of-life care. Embedding palliative-care teams in the ED to lead these discussions has shown promise in supporting both patients and physicians.^19^, ^20^

## LIMITATIONS

This study has several limitations. First, although our sample included physicians from both a quaternary-care center and several community hospitals, all participants practiced within a single, geographically expansive, and relatively lightly populated state where most of the population resides in a single urban area. This limits generalizability to geographic regions with large catchment areas for quaternary- care centers, where inter-hospital transport times may vary significantly. Because most community hospitals in our study were independent, our findings may not apply to systems where hospitals share ownership. While rural EDs were represented, EDs in frontier regions of the state, which may face even greater resource constraints, were not.

Additionally, participation was voluntary, introducing potential selection bias if individuals with stronger opinions were more likely to participate. All interviewers came from the quaternary-care center. While this may have introduced bias among referring physicians, we do not have data to determine its direction or magnitude. Finally, as this was a qualitative study, there was a risk of researcher interpretation bias. Efforts were made to mitigate this potential bias by involving multiple analysts and reconciling discrepancies during coding and thematic analysis. Researcher positionality was included to frame potential bias.

## CONCLUSION

This study provides a foundation for further work examining transfer decisions for potentially moribund patients. To improve the generalizability of these findings, further studies should re-create this study in other regions and other hospital systems. Future research should prospectively evaluate how structured pre-transfer goals-of-care discussions impact patient health outcomes, family satisfaction, and system resource use. Additionally, pilot-testing and evaluation of statewide guidelines and decision-support tools that assist with transfer decisions while preserving clinical judgment and flexibility may also provide value. However, as one participant noted, standardization may not always be the appropriate goal and could be counterproductive in certain circumstances.

It is also important to note that the patient’s and family’s voices are missing from this research; proactively incorporating input from patients and their families at all points (pre-transfer, immediate post-transfer, long-term follow-up) will be essential. Integrating these strategies into practice could help improve transfer practices for critically ill patients across diverse healthcare settings.

## Supplementary Information





## Figures and Tables

**Table:** Summary of themes in a qualitative study of the considerations of physicians transferring moribund patients from a community hospital and physicians accepting transfers to their quarternary-care center.

Theme	Representative summary	n (%) Accepting physicians reporting	n (%) Referring physicians reporting
Accepting physician’s perceived obligation to hospitals with fewer resources	Resource availability and the role of accepting hospitals within the state’s hospital ecosystem	8/8 (100%)	NA
Difficulty of prognosticating outcomes	Uncertainty about prognosis even in severe injury or illness predisposed physicians to transfer	6/8 (75%)	7/8 (88%)
Limitations of current advance care planning documents	Existing advance care planning documents were insufficient during major status changes or when family preferences or assertions diverged from what had been written.	6/8 (75%)	6/8 (75%)
Impact of patient and family preferences	Family wishes influenced transfer decisions.	5/8 (63%)	8/8 (100%)
