# Supplementary material for: Obligated To Say “Yes”: The How and Why Behind Transfer Decisions in Moribund Patients
Source: West J Emerg Med. 2026 Mar 2;27(2):236–43. doi: 10.5811/westjem.48985 (PMC13016042; doi:10.5811/westjem.48985)
Supplement: Supplementary file 1 [file wjem-27-236-s001.docx]

**Appendix A**

**ED Transfer Decisions Interview Guide - Referring Physicians Version**

*Background on the project*: The goal of this study is to better understand the reasoning and experience of physicians when requesting a transfer to a tertiary care center for a patient with potentially non-survivable injuries.

Do you have any questions before we get started?

1. Background information: Tell me about your training background, practice experience, and the resources / capabilities of the emergency department you currently work in (e.g. setting of hospital, available physical and human resources). Do you mean like cath lab, trauma, subspecialty services? What do you mean by physical and human resources?
   1. How challenging is it to transport patients to a tertiary care center in terms of EMS transport?
2. Can you talk me through your decision-making process for transferring patients with potentially non-survivable injuries/disease to tertiary hospitals?
   1. How do you talk to patients and families about prognosis prior to transfer in the setting of potentially non-survivable injuries/disease processes?
   2. How do family / patient preferences for transfers impact your decision making in these settings?
   3. How do the actual transfer logistics and predicted time (e.g. helicopter / ambulance / fixed wing) weigh into your decision making?
3. How do you factor in pre-existing code status/POLST when initiating a transfer for a potentially non-survivable problem?
   1. In your opinion/experience, when is it appropriate to reconsider code status prior to transfer?
4. If the decision was made to keep a patient with a potentially non-survivable injury / disease process at your hospital, rather than transfer, what would be the impact on your hospital / personnel?
   1. What additional resources / support would be needed?
5. What are your thoughts about a statewide transfer protocol that provides guidelines to assist with transfer decision making?
6. Anything else you feel is important to share?
