# Supplementary material for: Obligated To Say “Yes”: The How and Why Behind Transfer Decisions in Moribund Patients
Source: West J Emerg Med. 2026 Mar 2;27(2):236–43. doi: 10.5811/westjem.48985 (PMC13016042; doi:10.5811/westjem.48985)
Supplement: Supplementary file 2 [file wjem-27-236-s002.docx]

**Appendix B**

**ED Transfer Decisions Interview Guide - Accepting Physicians Version**

*Background on the project*: The goal of this study is to better understand the reasoning and experience of physicians when accepting a transfer to a tertiary care center for a patient with potentially non-survivable injuries.

Do you have any questions before we get started?

1. Background information: Tell me about your background and practice experience and the resources / capabilities of the emergency department you currently work in (e.g. setting of hospital, available physical and human resources).
   1. What EMS resources are available in your area?
2. Can you talk me through your decision-making process for accepting transfers of patients with potentially non-survivable injuries/disease processes to your hospital?
   1. How does family/patient preference or known goals of care impact your decision making in these settings?
   2. How do you factor in pre-existing code status/POLST when accepting a transfer for a potentially non-survivable problem?
      1. (If not included in answer to above question) Is checking and confirming code status a part of your transfer routine?
   3. How do the actual transfer logistics (e.g. helicopter / ambulance / fixed wing) weigh into your decision making?
3. At what point do you elect to involve your neurosurgical or trauma surgery teams in a pre-transfer conversation?
4. Can you tell me about a time that you or a colleague had to refuse a transfer and why that decision was made?
5. What are your thoughts about a statewide transfer protocol that provides guidelines to assist with transfer decision making?

Anything else you feel is important to share?
